# Communication as teaching content of veterinary studies – a joint position paper from the DVG specialist group “communication and didactics” and the GMA veterinary medicine committee

**DOI:** 10.3205/zma001480

**Published:** 2021-04-15

**Authors:** Christian Gruber, Marc Dilly, Mahtab Bahramsoltani, Christin Kleinsorgen, Simon Engelskirchen, Sabine Ramspott, Jan P. Ehlers

**Affiliations:** 1 veted-consulting by Christian Gruber, Munich, Germany; 2 scil vet academy, Viernheim. Germany; 3 Justus-Liebig-University Giessen, Faculty of Veterinary Medicine, Giessen, Germany; 4 Freie Universitaet Berlin, Institute of Veterinary Anatomy, Berlin, Germany; 5 University of Veterinary Medicine Hannover, Center for E-Learning, Didactics and Educational Research, Hannover, Germany; 6 AniCura Duisburg-Asterlagen GmbH, Duisburg, Germany; 7 Trillium GmbH Medizinischer Fachverlag, Grafrath, Germany; 8 University of Witten/Herdecke, Faculty of Health, Chair of Didactics and Educational Research in Health Care, Witten, Germany

**Keywords:** communication, veterinary medicine, communication competence

## Abstract

Veterinarians have to face many communicative challenges in their professional capacity. Successful professional communication increases satisfaction among pet owners, staff members and colleagues, and ultimately also the veterinarians’ own satisfaction. On the other hand, unsuccessful communication can easily lead to rejection, distrust and dissatisfaction.

However, communicative skills are not explicitly taught as part of the compulsory courses in veterinary medicine in Germany.

The position paper of the GMA Veterinary Medicine Committee and the DVG work group Didactics and Communication Competence describes the importance of successful communication for working veterinarians as well as the training situation in Germany and addresses topics that are often discussed in connection with the implementation.

The authors come to the conclusion that there is both a necessity and a possibility for the introduction of communicative training content and provide recommendations that are intended to support the sustainable introduction of courses and exams to develop the communicative skills of veterinary students.

## Zusammenfassung

Tierärzt*innen müssen sich in der Ausübung ihres Berufes vielen kommunikativen Herausforderungen stellen. Gelungene professionelle Kommunikation führt dabei zur Förderung der Zufriedenheit bei Patientenbesitzer*innen, Mitarbeiter*innen, Kolleg*innen, sowie letztlich auch zur Steigerung der eigenen Zufriedenheit. Misslungene Kommunikation kann im Gegenteil leicht zu Ablehnung, Misstrauen und Unzufriedenheit führen.

In den Pflichtveranstaltungen im Studium der Tiermedizin in Deutschland findet jedoch keine explizite Vermittlung kommunikativer Fertigkeiten.

Das Positionspapier des GMA-Ausschusses „Tiermedizin“ und des DVG-Arbeitskreises „Didaktik und Kommunikationskompetenz“ beschreibt die Bedeutung gelungener Kommunikation für das Berufsleben von Tierärzt*innen sowie die Ausbildungssituation in Deutschland und greift Themen auf, die im Zusammenhang mit der Implementierung oftmals diskutiert werden.

Die Autor*innen kommen zu dem Schluss, dass sowohl die Notwendigkeit als auch die Möglichkeit für die Einführung von Ausbildungsinhalten aus dem Bereich der Kommunikation gegeben sind und stellen Empfehlungen zur Verfügung, die eine nachhaltige Einführung von Lehrveranstaltungen und Prüfungen zur Entwicklung der kommunikativen Fertigkeiten der Tiermedizinstudierenden unterstützen sollen.

## Introduction

In the everyday professional life of many people, including (veterinary) medical professionals, communication is an essential factor that not only has a significant influence on the quality of work, but also on job satisfaction [22], [31], [32].

Overall, however, (veterinary) medical professionals are expected to have above-average communication skills, for instance in dealing with patients and – in the case of veterinary medicine – their owners, but also within teams and among colleagues. For this reason, veterinarians need communicative skills in many areas in order to be successful in their profession, as the arbitrarily compiled selection of communication roles and situations shows (see table 1 (Tab. 1)). 

Despite the apparently far-reaching importance of successful communication for professional development, there has not yet been any explicit teaching of professional communication skills in the compulsory part of the veterinary curriculum in Germany.

## Objective of the position paper

The aim of this position paper is to present the importance of successful communication for the professional success of veterinarians. The need for structured training in communication for veterinary students is derived from this.

Central topics that can play a role in the implementation of communication training are addressed and result in general recommendations that are intended to serve as a guideline for sustainable implementation.

## Development process

Following a one-day workshop in January 2015 on the subject of “Communication in Veterinary Studies”, to which the Veterinary Medicine Committee of the Society for Medical Education (GMA) and the Work Group for Didactics and Communication Competence of the German Veterinary Medical Society (DVG) invited their members, four groups were formed for the next stage of the process:

1. the status quo of communication training in veterinary medicine in Germany,
2. literature and additional sources for communication in (veterinary) medicine,
3. learning objectives and learning outcomes for a sample curriculum,
4. organization of further events (this group was merged with Group 2 because in due course all members of this group (Head of the DVG work group and the GMA committee) were also members of group 2).

The work carried out by these groups served as the starting point for this position paper. Communication between the members of all four groups took place in a closed group on a veterinary platform [1] and in several online meetings using Adobe Connect^®^. During the development process of the position paper, the members of the other groups and interested members of the two committees also had the opportunity to access the respective drafts. The present selection of literature, topics and recommendations was developed by the authors in a consensus process [37]. 

The position paper was submitted to the DVG work group and the GMA Committee for comment and subjected to peer review by the GMA.

## Results

The importance of successful communication for professional success

As a basis for professional success in veterinary practices measured by income, three key management criteria were described in addition to technical expertise [30]:

- long-term members of staff,
- staff satisfaction and
- customer satisfaction (=pet owners).

Compassion, honesty and trustworthiness are among the most important factors for the satisfaction of pet owners, especially in veterinary medicine [5]. In addition, the perception of professional and competent appearance leads to an increase in trustworthiness, as was found in a study with students and standardized pet owners [14]. All of these points are based on good communication, and even personal job satisfaction correlates with good communication skills [3]. 

On the other hand, a lack of communication skills is a notable and noticed source of unprofessional behavior. A survey of complaints received between 2002 and 2004 at the College of Veterinarians of Ontario [34] shows that 60-67% of problems related to deficits in the area of communication. Deficits in the area of communication and inappropriate behavior can even lead to pet owners developing a personal dislike of the veterinary profession as a whole [24]. Accordingly, communication is also a fundamental skill for good veterinary medicine practice (GVP) and as such is explicitly written down in the GVP code [8]. 

Due to the great influence of communication on professional success in medical professions in general, it seems obvious to prepare the students for this important part of their later work during their studies. On the one hand, to establish and impart state of the art communication that is appropriate for the profession, and on the other hand to meet the obligation of educational institutions to turn out not only professionally qualified graduates but such who are ready for the workplace. 

### Communication as part of veterinary training

According to the requirements of the European Association of Establishments for Veterinary Education (EAEVE), which apply across Europe, veterinarians should have certain general skills to practice their profession – the so-called day one competences – when they have completed their veterinary training [11]. The following day one competences are directly or indirectly related to communication (see table 2 (Tab. 2)).

In addition, the EAEVE 2016 added the subject Professional Communication to the list of subjects that are considered to be part of (basic) veterinary training [11]. 

The EAEVE is thus following an international trend that developed, among other things, following studies that demonstrated that the teaching of communication skills in veterinary training had not been given sufficient emphasis [6], [20]. 

#### Communication training in veterinary studies in Germany

The Veterinary Licensure Act (TAppV) defines the framework for the curricula of all five German veterinary training institutions. Veterinary training includes a scientific-theoretical part of 3,850 hours and a practical study part of 1,170 hours. By the end of the five and a half year course, students have to take exams in a total of 29 examination subjects [https://www.gesetze-im-internet.de/tappv/BJNR182700006.html]. The TAppV does not currently have a separate examination subject for communication. Only some exam content in some subjects indicates the presence of communicative components in teaching (e.g. “explaining treatment plans”). It is often argued that training takes place implicitly as part of the clinical practical courses. In addition, some veterinary educational institutions have started to offer elective courses (for a small number of students) on the subject of communication [3].

Although the TAppV does not explicitly mention the word communication, examination content is named in several examination subjects that clearly require communicative competencies, such as

§ 44 (General Pathology and Special Pathological Anatomy and Histology): “... determine and explain pathological-histological specimens, ... explain the findings and then write them down ...”

§ 48-50 (Reproductive medicine, Internal medicine, Surgery and Anesthesiology): “... to design and explain a treatment plan..., ... and to create a written report of findings on an examined animal.” [https://www.gesetze-im-internet.de/tappv/BJNR182700006.html].

The requirement for verbal and written communication skills in a professional context is only expressed here as part of some subject descriptions, but one could derive from this an obligation to include these communication skills explicitly in compulsory teaching and to test them appropriately, for example in courses which are compulsory for admission to the examination or as part of the so-called cross-sectional teaching according to § 53 TAppV.

#### Different countries, different training courses – international examples

In Austria and Switzerland [35], [36] there are explicit teaching/learning objectives from the field of communication in the curricula. In the Netherlands, communication is listed separately as an independent skill in the Competency Framework [4]. There are also explicit teaching and competency goals for communication in the curricula for veterinary training in English-speaking countries, for instance in North America [34]. 

A look beyond the veterinary horizon – training in communication in the human health professions and for veterinary specialist staff

With the introduction of the CanMeds roles at the very latest [13], in which the various roles that doctors (must) perform in the exercise of their profession are dealt with, the roles of communicator and collaborator listed therein have led to these areas penetrating the consciousness of those responsible for training as necessary training content – first in postgraduate training, then also in basic studies. In the German-speaking countries, a comprehensive catalog with social and communication skills for medical studies was compiled in the Basel Consensus Statement in 2008 via a broad process [17]. Since 2015, learning objectives and learning outcomes in the area of social competences and communication have been anchored in the National Competence-Based Catalog of Learning Objectives for Medicine and Dentistry [12]. Although communication is not mentioned as training content in the Medical Licensure Act of 2002, just as it is not mentioned in the TAppV, the doctor-patient relationship as well as medical communication is an integral part of training at medical training facilities in the medical curricula, for example in the subject of medical psychology [16]. 

Even in the non-academic field of veterinary medicine, i.e. among veterinary specialist staff, communicative competence is explicitly anchored as a training goal [https://www.gesetze-im-internet.de/tiermedfangausbv/BJNR252200005.html]. Various forms of communication and communication methods for advising and dealing with pet owners must be learned, as well as behavior in conflict situations. 

The question that has to be raised here is the extent to which veterinarians who are not trained in communication are able to guide the veterinary specialist staff employed by them in questions of communicative competence.

## Central topics in the implementation of communication training

The following topics are often discussed when considering the importance of communication in veterinary training:

### Implicit teaching of communication through social learning 

We speak of implicit learning when the behavior of learners who have taken a learning unit follows new regular patterns without these being conveyed by the teacher or the learners consciously realizing them [26]. Implicit learning is individual and non-selective. Brown and Bylund (2008) recommend a mixture of explicit (learning objectives, teaching, feedback) and implicit learning (internships, observations) for teaching communication skills in the medical field [7]. It is important that implicit knowledge and behavior are made explicit in order to be able to use the skills successfully [2], [38]. 

A study at the University of Veterinary Medicine in Hanover shows that students who were only implicitly trained in communication skills achieved far worse results in an objectively structured clinical test than students who had taken part in communication training [10]. The common assumption that the area of communication and especially the interaction between veterinarians and pet owners is implicitly conveyed in the clinical examination subjects through more or less active participation in conversations in hospitals or during internships and learned by students therefore is not correct. In addition, there are no explicit criteria which could guide teachers and learners regarding the required standards of communicative skills. The result is that it is neither summarily nor formatively possible to check and evaluate the level of the communicative skills of the students and graduates.

#### Time allocation in the curriculum

The introduction of new learning content in a time-capped curriculum requires the reallocation of time resources.

On the one hand, it could be strictly argued that the compulsory subjects, which in the TAppV have a communicative part, must also spend a manageable part of their compulsory hours quota for the explicit training of communicative skills. On the other hand, it could be easier, in terms of university administration, to argue for moving lessons from the compulsory elective subject area to the compulsory hours quota or to combine these two measures.

Ultimately, however, the revision of the existing and the adaptation to a changing environment cannot be ignored by veterinary training institutions, even if one only considers the professional development of the subject itself. As a scientific discipline, communication does not belong to any subject area that could originally be assigned to veterinary medicine, which has certainly been detrimental to its inclusion in the compulsory teaching of veterinary medicine. Given the importance of communication in exercising their role as veterinarians and from the point of view that a degree course in veterinary medicine is the sole path to becoming a veterinarian, communication is a key competence that should in no way be inferior to the other training content in the curriculum.

#### Evaluation of communicative skills

There is already a great deal of literature on this topic, especially in human medicine, to which reference is made in this context. For example, the European Association for Communication in Healthcare (EACH) has published so-called General Principles for the Assessment of Communication Skills in which numerous methods for testing communicative competencies and their areas of application are listed [18].

In addition to written examination methods, there are also other examination methods such as OSCEs, virtual and standardized patients, portfolios, mini-CEX or 360° feedback. All of these procedures are already widespread and well-established, so that the innovation effort would only be in the translation into the veterinary context.

In addition to the selection of the examination formats, the content of the examination criteria is often seen as a (too) big challenge. For human medicine, a comprehensive catalog of communicative and social skills in medical studies has already been presented in the Basel Consensus Statement [17]. In addition, several consultation models for doctor-patient discussions were developed and implemented in medical training [9], [19], [25], [27]. The content-related proximity to human medicine suggested translation of the models into the veterinary context would be possible, and accordingly they were adapted and introduced for discussions with pet owners [15], [21], [23], so that a broad basis of practical examples already exists here as well.

#### Training and qualification of the teaching staff

The implementation of the subject communication requires a university and subject didactic qualification of the lecturers in this area. Supposedly, this is associated with a high expenditure of time and money for both the lecturers and the educational institutions.

However, within the scope of quality assurance in veterinary teaching, there is already a series of higher education didactic training courses for lecturers, especially as didactic qualifications are prescribed in the post-doctoral rules and appointment regulations of some veterinary educational institutions. Teaching the necessary skills to the teaching staff could easily take place in these advanced training courses, which are increasingly common. 

In order to limit personnel expenditure, especially initially when introducing the corresponding courses, cooperation with human medicine and psychology at the respective location has also proven itself as an approach [16], especially as part of a “train the trainer” concept to train and qualify their own teaching staff step by step.

#### Engagement with students

Ultimately, the main target of every training measure are the students, which is why their uptake of the planned innovation is not negligible. In a qualitative study from England, students of human medicine had a partly positive and negative attitude towards communication skills training. In the case of students with a negative attitude towards communication training, this was based on their view that training in communicative competence was “not scientific enough”. The students with a positive attitude considered communicative skills to be important for professional success [28]. 

In veterinary medicine, in a qualitative survey on the requirements for a center for clinical skills, the participating students, lecturers and practicing veterinarians wished for, among other things, training in basic handling of pet patients and their owners, communication skills and team skills. In addition students in the 6th semester indicated they were least prepared to deal with pet owners and they wished for opportunities to practice during their studies in order to be able to communicate with pet owners in a more comprehensible and targeted manner. Students who had already taken part in courses on communication with pet owners (including role-playing) stated that they saw this form of preparation as essential and criticized the previous, in their view, insufficient teaching of these skills [29].

Ultimately, however, it was also possible to show that communication training during the course actually led to an improvement in the communicative behavior of the students. This also meant that pet owners were better able to remember what was said [21].

## Recommendations

The importance of good communication for professional success and the examples from other countries and related professions formed the basis for the development of this position paper by the GMA veterinary medicine committee and the DVG’s didactics and communication work group. For the authors, based on the above, not only the necessity but also the feasibility arises for the explicit introduction of communication teaching content in the compulsory parts of basic veterinary training.

From the point of view of the two committees, the following points should therefore be taken into account when designing future requirements for veterinary training:

- A minimum standard for communication training should be anchored in the TAppV so that training is integrated into compulsory teaching and examinations.
- Learning objectives and learning outcomes respectively on the subject of communication must be explicitly stated in the curricula.
- These learning objectives or learning outcomes must be part of integrative exams.
- In addition to lectures to introduce the topic, seminars and exercises in particular are to be provided, as it has been shown that building communication skills with role-play and subsequent feedback is more effective than through purely lecture-based teaching [15].
- The integration of the learning objectives or learning outcomes on the subject of communication in other subjects and teaching/learning events corresponds to the cross-sectional character of the subject, but requires that the teachers, as part of personnel development, are trained and qualified to guide and check the students in acquiring skills.

## Notes

The position paper was accepted by the GMA executive board at 02-16-2021.

## Acknowledgements

The position paper owes its existence to many people beyond the authors. We would like to thank everyone who supported and accompanied this project with their interest and commitment, namely (alphabetically, without title):

Christina Beitz-Radzio, Dora Bernigau, Astrid Bienert-Zeit, Corinna Eule, Silke Post (born Gaida), Thekla Großböhmer, Joachim Lübbo Kleen, Susan Kopke, Peter Stucki, Andrea Tipold. 

Special thanks go to the two reviewers from the GMA, Anja Härtl and Claudia Kiessling, for their valuable advice.

## Competing interests

The authors declare that they have no competing interests. 

## Figures and Tables

**Table 1:**
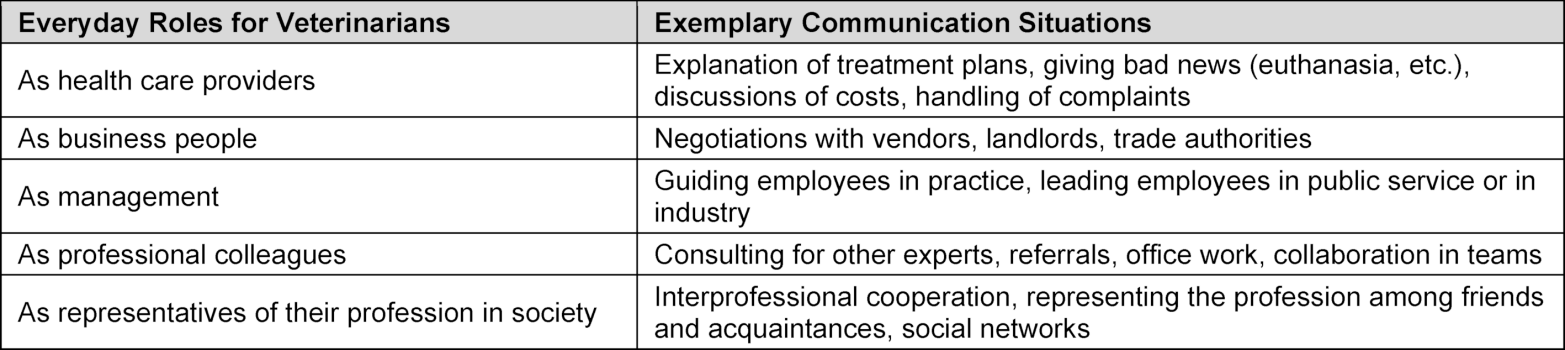
Examples of communication roles required of veterinarians

**Table 2:**
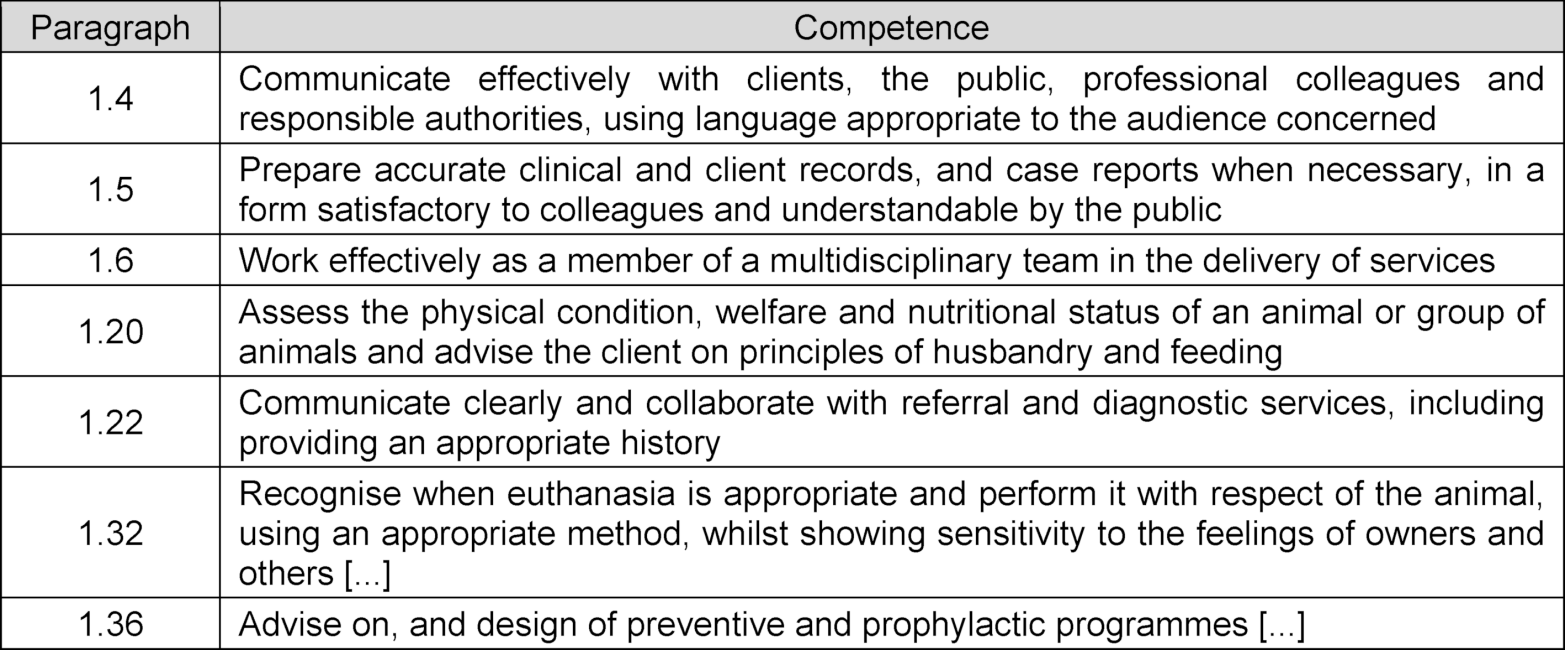
EAEVE Day One Competences related to communication
